# Impact of poverty and family adversity on adolescent health: a multi-trajectory analysis using the UK Millennium Cohort Study

**DOI:** 10.1016/j.lanepe.2021.100279

**Published:** 2021-11-30

**Authors:** Nicholas Kofi Adjei, Daniela K. Schlüter, Viviane S. Straatmann, Gabriella Melis, Kate M. Fleming, Ruth McGovern, Louise M. Howard, Eileen Kaner, Ingrid Wolfe, David C. Taylor-Robinson, ORACLE consortium

**Affiliations:** a Department of Public Health, Policy and Systems, University of Liverpool, Liverpool United Kingdom; b Department of Public Health Sciences, Stockholm University, Stockholm Sweden; c Population Health Sciences Institute, Newcastle University, Newcastle United Kingdom; d Department of Health Service and Population Research, King's College London, London, United Kingdom; e Department of Women and Children's Health, King's College London, London, United Kingdom

**Keywords:** child poverty, family adversity, child health, cohort, multi-trajectory analysis

## Abstract

**Background:**

Children exposed to poverty and family adversities including domestic violence, parental mental ill health and parental alcohol misuse may experience poor outcomes across the life course. However, the complex interrelationships between these exposures in childhood are unclear. We therefore assessed the clustering of trajectories of household poverty and family adversities and their impacts on adolescent health outcomes.

**Methods:**

We used longitudinal data from the UK Millennium Cohort study on 11564 children followed to age 14 years. Family adversities included parent reported domestic violence and abuse, poor mental health and frequent alcohol use. We used a group-based multi-trajectory cluster model to identify trajectories of poverty and family adversity for children. We assessed associations of these trajectories with child physical, mental and behavioural outcomes at age 14 years using multivariable logistic regression, adjusting for confounders.

**Findings:**

Six trajectories were identified: low poverty and family adversity (43·2%), persistent parental alcohol use (7·7%), persistent domestic violence and abuse (3·4%), persistent poor parental mental health (11·9%), persistent poverty (22·6%) and persistent poverty and poor parental mental health (11·1%). Compared with children exposed to low poverty and adversity, children in the persistent adversity trajectory groups experienced worse outcomes; those exposed to persistent poor parental mental health and poverty were particularly at increased risk of socioemotional behavioural problems (adjusted odds ratio 6·4; 95% CI 5·0 – 8·3), cognitive disability (aOR 2·1; CI 1·5 – 2·8), drug experimentation (aOR 2·8; CI 1·8 – 4·2) and obesity (aOR 1·8; CI 1·3 – 2·5).

**Interpretation:**

In a contemporary UK cohort, persistent poverty and/or persistent poor parental mental health affects over four in ten children. The combination of both affects one in ten children and is strongly associated with adverse child outcomes, particularly poor child mental health.

**Funding:**

The National Institute for Health Research (NIHR) Policy Research Programme, NIHR Applied Research Collaboration South London (ARC South London) at King's College Hospital NHS Foundation Trust and the Medical Research Council (MRC).

**Research in context**

**Evidence before this study**

We systematically searched MEDLINE, PsycInfo, and the Web of Science for articles published up to March 15, 2021, without language restrictions for studies that assessed the associations between family adversity (measured using poor parental mental health, domestic violence and abuse and parental alcohol use), poverty and child health outcomes in adolescence with the search terms provided in the appendix· Our search yielded fewer than 10 studies. We found that these studies have mainly focused on the effects of single adversities and have not looked at the long-term patterns of dynamic trajectories of multiple family adversities. No studies in our search investigated the clustering of trajectories of child poverty and family adversities and their impacts on adolescent health outcomes at age 14.

**Added value of this study**

We used a multi-trajectory approach applied to rich nationally representative birth cohort data from the UK Millennium Cohort Study to assess the clustering of household poverty and family adversities and their impacts on child health outcomes in adolescence. To our knowledge, this is the first study to assess the impact of multiple family adversities and household poverty across childhood. Our result shows that over 40% of children experienced continuous exposure to either poverty and/or poor parental mental health and these common exposures are associated with large negative impacts on child physical, mental, cognitive and behavioural outcomes.

**Implications of all available evidence**

Our findings suggest that interventions to address specific childhood adversities such as parental mental health problems may not be meaningful if childhood socioeconomic conditions such as poverty are not considered. Parental mental health problems interact syndemically with structural risk factors such as poverty across childhood developmental stages, with large negative impacts on health outcomes and behaviour in later life. Policies that address upstream drivers of poor child health, and tackle the synergistic interaction of two or more coexisting risk problems, may ameliorate adverse health and behavioural outcomes in children and adolescents.

Alt-text: Unlabelled box

## Introduction

Adverse childhood experiences (ACEs) such as abuse, neglect and family adversities are pressing public health issues^1^ garnering policy attention in many countries.^2^^,^^3^ Previous studies on ACEs have established that family adversity in particular (parental mental illness, domestic violence and abuse and alcohol use) increases the risk to children's welfare and safety.^4^ In the UK, approximately one in four children aged 0-16 years live with a parent affected by mental health problems.^5^ A recent review of cohort studies showed that between 5% and 18% of UK children aged 9-12 months to 14 years have lived with a parent affected by increased risk of alcohol misuse.^6^

Childhood adversities are known to co-occur or cluster together,^7^, ^8^, ^9^ and there is some evidence that poverty is a strong reinforcing factor in the clustering and accumulation of adversity.^7^ In the UK, for instance, around one in three children is currently in relative income poverty and it is estimated that one in every five children is at risk of persistent poverty^10^ Children growing up in poverty are more likely to experience clusters of adversities,^7^ and to experience adverse outcomes across the life course, including developmental delays,^11^ injuries,^12^ physical and mental ill health,^13^ poor educational outcomes,^14^ chronic health conditions,^9^ and premature mortality.^8^

Despite the substantial evidence that childhood adversities influence family functioning are closely linked with poverty and material deprivation,^7^^,^^9^ the dynamics of these exposures across childhood developmental stages are less clear. Furthermore, socioeconomic conditions (SECs) such as poverty and other family level childhood adversities are frequently conflated.^9^ Since poverty may be an important structural risk factor for and consequence of other childhood adversities,^7^ disentangling their interactive effects over time is challenging, but important in order to develop appropriate public health strategies and interventions.^15^

As a first step, isolating clusters of children who share common trajectories of poverty and other family adversities across the life course may be helpful to clarify population strata that may benefit from particular interventions. Novel methods such as the group-based multi-trajectory modelling have recently been used to identify trajectories of childhood adversity in Denmark^8^ and exposure to poverty in the UK. Using data from a large cohort in the UK, we explored child-level prevalence for exposure to family adversity and child poverty over time. We then aimed to assess the clustering of trajectories of child poverty and family adversities and their impacts on subsequent child behaviour and reported health outcomes in adolescence.

## Methods

### Study design and population

We used data from the Millennium Cohort Study (MCS), a large nationally representative cohort sample of British children born between September 2000 and January 2002 and followed up through six survey waves, when the children were 9 months, 3, 5, 7, 11 and 14 years of age. The wave 6 (age 14 years) data were collected from January 2015 to March 2016 when the cohort children were in secondary school. At each wave, information on the children was collected from the main carer, usually the child's mother (about 99% at wave 1, 96% by wave 6). Unless otherwise specified, references to parents, such as poor parental mental health refers to the main carer. The numbers of responding families at waves, 1, 2, 3, 4, 5 and 6 were 18 552, 15 590, 15 246, 13 857, 13 287 and 11 726, respectively. We did not do a formal sample size calculation. We used data from the birth survey (wave 1) and follow-ups, and included only singletons (i.e., not twin or other multiple pregnancies) in our analysis. The MCS oversampled children living in high-deprivation areas and those belonging to high minority ethnic populations by means of a stratified cluster sampling. Further information regarding survey design and sampling is detailed elsewhere.^16^ The data collection of Millennium Cohort Study (MCS) is approved by the UK National Health Service Research Ethics Committee and written consent was obtained from all participating parents at each survey; MCS1: South West MREC (MREC/01/6/19); MCS2 and MCS3: London MREC (MREC/03/2/022, 05/MRE02/46); MCS4: Yorkshire MREC (07/MRE03/32); MCS5: Yorkshire and The Humber-Leeds East (11/YH/0203); MCS6: London MREC(13/LO/1786). The present analyses did not require additional ethical approval.

## Measures

### Components of exposure trajectories: poor parental mental health, frequent alcohol use, domestic violence and poverty

The main exposures were trajectories family adversity and poverty from age 9 months to 14 years. Longitudinal measures of exposure were created from the indicators of family adversity of interest (i.e., poor parental mental health^17^^,^^18^, domestic violence and abuse, and frequent parental alcohol use) and poverty^10^ from 9 months to age 14 years. A binary score was constructed for all exposures throughout childhood^10^^,^^19^ (for full details see Box 1).

Box 1 **Description of measurements assessed for trajectory exposures**

▪ **Poor parental mental health (Child aged 9 months)** – Rutter Malaise Inventory (RMI)^16^ scale was used to assess parental mental ill health in the last 30 days· A shortened 9-item self-completed version of the RMI measuring depression, anxiety and psychosomatic illness was used· The 9-item short form included items ‘feel tired most of the time’, ‘feel miserable or depressed’, ‘worried about things’, ‘often get into violent rage’ ‘suddenly become scared for no good reason’, ‘easily upset or irritated’, ‘constantly keyed up or jittery’, ‘every little thing gets on nerves and wears you out’, and ‘heart race like mad’· Scores from these items were summed, and we used a validated cut off for mental ill health [‘yes (scores >=4)/no’]·

▪ **Poor parental mental health (Child aged 3 to 14 years) –** Kessler 6 (K6)^17^ scale was used to assess parental mental ill health in the last 30 days asking the responders how often they felt depressed, hopeless, restless or fidgety, worthless, or that everything was an effort· Respondents answered on a five-point scale from 1(all the time) to 5 (none of the time)· We reversed and rescaled all items from 0 to 4 for analysis purposes, so that high scores indicate high levels of psychological distress· We used a validated cutoff widely used in previous studies [‘yes (scores >=6)/no’]^22^

▪ **Frequent parental alcohol use (Child aged 9 months to 7 years) –** the main responder responded a question about the usual frequency of alcohol consumption (‘*Every day, 5-6 times per week, 3-4 times per week, 1-2 per week, 1-2 per month, less than once a month or never’*)· Dichotomised: [every day and 5-6 times per week (Yes) vs· 3-4 per week/1-2 per week/ 1-2 per month/never (No)]^18^

▪ **Frequent parental alcohol use (Child aged 11 to 14 years) –** the main responder responded a question about the usual frequency of alcohol consumption *(‘>=4 times per week, 2-3 times per week, 2-4 times per month, monthly or less, or never’)·*Dichotomised: [4 or more times a week (Yes) vs· 2-3 per week/2-4 per month/ monthly or less/never (No)]^18^

▪ **Domestic violence and abuse (Child aged 9 months to 14 years) –** the main responder was questioned about whether “husband or partner ever used physical force in the relationship” (‘*Yes, No’*)^18^

▪ **Poverty (Child aged 9 months to 14 years)** – relative income poverty^4^, defined as household equivalised income of less than 60% of national median household income equivalised* according to the Organisation for Economic Co-operation and Development (OECD) household equivalence scale.

*Equivalised” means that the Organisation for Economic Co-operation and Development (OECD) household equivalence scales were applied to net income figures, which takes into account the number and age of adults and dependents in the household, giving a more accurate representation of a household's available resources relevant to its size and composition. This equivalised income measure is commonly used in studies of poverty in the UK, including using MCS^9^

Alt-text: Unlabelled box

### Outcomes

The main outcomes were socioemotional behavioural problems, cognitive disability, obesity, and alcohol and drug experimentation at age 14 years. Child socioemotional behavioural problems were assessed using the Strengths and Difficulties Questionnaire (SDQ) completed by the parent/main caregiver. The SDQ is a 25-item measuring five scales: hyperactivity, emotional symptoms, conduct disorders, peer problems and prosocial behaviour. We used the total difficulties score (excluding prosocial behaviour items) to classify children in two groups using validated cut-offs:^20^ ‘normal to borderline behaviour problems’ (0-16), and ‘socioemotional behavioural problems’ (17-40). The internal consistency of this measure was good in the study sample (Cronbach's α=0.77). Cognitive (dis)ability was assessed through the word activity test, which measures understanding of the meaning of words. The test has 20 different words in English and five possible synonyms for each word, and children were required to match each word with the correct synonym. We applied a widely used validated cut-off score^19^^,^^21^ of -1.25 standard deviation (SD) below the normed mean score to define children as having cognitive disabilities. Obesity was derived from the body mass index (kg/m^2^) of children, using the International Obesity Task Force age and sex specific BMI cut-offs.^22^ For health-related behaviours, measures of alcohol and drug experimentation were used. To assess alcohol experimentation at age 14, we used questions on whether the adolescent had ever had an alcoholic drink. Response categories were: ‘yes’ (coded as 1) or ‘no’ (coded as 0). For drug experimentation, whether the adolescent had ever tried cannabis (also known as weed, marijuana, dope, hash or skunk). Response categories were: ‘yes’ (coded as 1) or ‘no’ (coded as 0).

### Covariates

We considered child sex, maternal education (degree plus, diploma, A-levels, GCSE A-C, GCSE D-G, none), maternal ethnicity (white, mixed, Indian, Pakistani and Bangladeshi, black or Black British, or other ethnic groups) and lone parenthood when the child was aged 9 months as potential confounders^19^^,^^23^^,^^24^, guided by a directed acyclic graph (appendix, pp 2).

### Statistical Analysis

First, we estimated the cross-sectional child-level prevalence of parental mental ill health, domestic violence and abuse, frequent parental alcohol use, and poverty, singly and in combination, at child's age 9 months, 3, 5, 7, 11 and 14 years. Second, we used a group-based multi-trajectory model to determine trajectory groups of adversities from early childhood to late childhood. This approach identifies clusters of children who share similar trajectories of multiple exposures over time.^25^ In order to determine the trajectory groups that best fit the data, we fitted between one and seven trajectory clusters using logistic regressions with cubic trajectory functions of age (see supplementary material for more details on the model specification). This yields a probability for each child of being in each trajectory group at each age. We did not go beyond seven clusters to preserve theoretical coherence and parsimony^25^^,^^26^ given the sample size in the data. Following the suggestion by Nagin et al.,^26^ the models were selected based on the Bayesian information criterion (BIC) (appendix, pp 5). The BIC values closest to zero denote a better fitting model. The adequacy of the selected model was further judged by: (a) sufficient sample sizes in each identified trajectory group, (b) average posterior probabilities of assignment (AvePP > 0.70), and (c) odds of correct classification based on the posterior probabilities of group membership (OCC > 5.0) (appendix, pp 5). Individuals were assigned to the group having the highest posterior probability (i.e., using the maximum probability assignment rule). Furthermore, we qualitatively judged that the six trajectory groups divided the individuals optimally.^26^ The models were fitted with STATA 14.2 *TRAJ* package,^27^ and Full-information maximum likelihood (FIML) was used to account for missing data.^26^ Longitudinal weights were used to account for response bias and attrition.

Finally, we assessed the associations between adversity trajectories and our outcomes using logistic regression in a complete case analysis. (ORs) and 95% confidence intervals (CIs) were calculated. These models were also adjusted for child's sex, maternal education, maternal ethnicity and lone parenthood when the child was 9 months old. We conducted a number of additional analyses to test the robustness of our results. First, repeating the analysis using multiple imputation by chained equation (25 imputed data sets) with results pooled using Rubin's rules;^28^ second, repeating the analysis using the multiple pseudo-class draw method^29^ (20 draws) to account for uncertainty that may arise in group membership; third, assessing whether the associations between trajectory groups and child outcomes varied by child's sex by including interaction terms.

### Role of the funding source

The funders of the study had no role in study design, data collection, data analysis, data interpretation, or writing of the report. The corresponding author had full access to all the data in the study and had final responsibility for the decision to submit for publication.

## Results

### Study Population Characteristics

**Figure 1:**
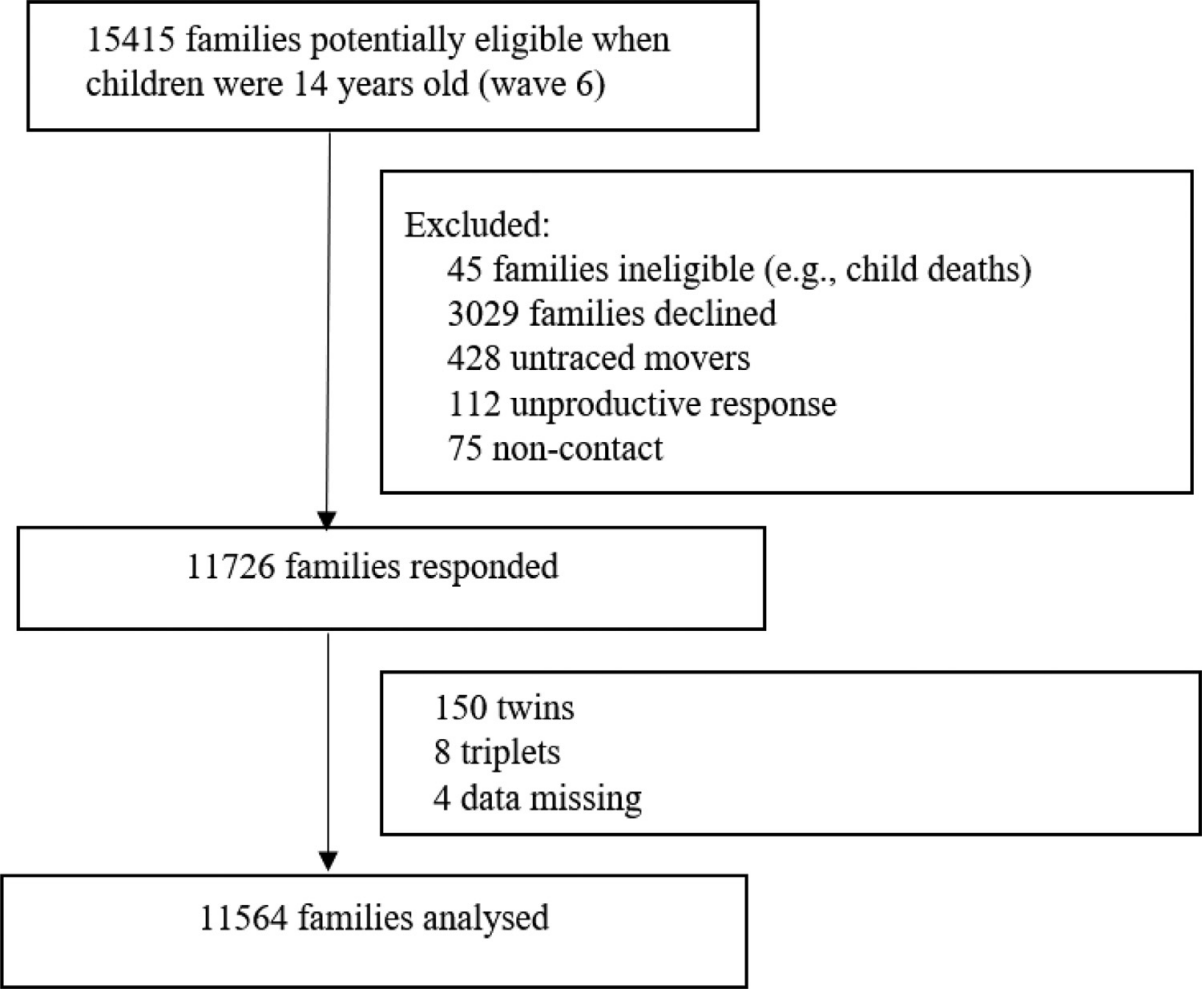
Study flow diagram showing inclusion and exclusion of cohort participant

Of the 15415 families who were eligible in the MCS at 14 years, 11564 families were analysed (Fig. 1). Table 1 shows the weighted estimated cross-sectional child-level prevalence of each family adversity measure, poverty and their combinations from early childhood to late childhood. Poverty was the most commonly experienced exposure, followed by family adversities: poor parental mental health, frequent alcohol use and domestic violence and abuse. The percentage of children in poverty increased from 30·3% at age 9 months to 34·6% at age 14 years. Parental self-reported mental ill health increased with child age from 13·6% at age 9 months to 32·1% at age 14 years. The percentage of children exposed to frequent parental alcohol use increased from 5·5% at age 9 months to 7·7% at age 14 years. Exposure to parental domestic violence and abuse was stable at around 3-4%. Less than 0·5% of children experienced all three family adversities at each age follow-up, with 0·14% experiencing all three adversities at age 14 years.

**Table 1:** Estimated child-level prevalence for family adversity measures (domestic violence and abuse, parental alcohol use, poor parental mental health) and poverty in the UK Millennium Cohort Study, weighted sample

	Age 9 months	Age 3	Age 5	Age 7	Age 11	Age 14
	%	%	%	%	%	%
Domestic violence and abuse	3.6 (3.2-3.9)	4.4 (4.0-4.8)	4.0 (3.6-4.4)	3.8 (3.4-4.2)	3.9 (3.4-4.4)	3.2 (2.7-3.7)
Parental alcohol use	5.5 (4.6-6.5)	6.7 (5.9-7.5)	7.8 (6.9-8.6)	7.4 (6.7-8.1)	8.3 (7.5-9.2)	7.7 (6.9-8.5)
Poor parental mental health	13.6 (12.9-14.3)*	19.0 (18.0-19.8)	18.8 (17.9-19.7)	20.0 (19.0-20.8)	28.3 (27.0-29.5)	32.1 (30.7-33.5)
At least 1 family adversity	19.7 (18.6-20.8)	25.1 (24.0-26.1)	24.8 (23.6-25.9)	25.5(24.4-26.5)	32.7 (31.4-34.0)	35.2 (33.7-36.6)
At least 2 family adversity	1.9 (1.6-2.1)	2.9 (2.5-3.2)	2.8 (2.4-3.2)	2.8 (2.4-3.1)	3.4 (2.9-3.9)	3.8 (3.2-4.4)
All 3 family adversities	0.08 (0.04-0.16)	0.11 (0.05-0.22)	0.18 (0.10-0.31)	0.17 (0.09-0.29)	0.24 (0.14-0.39)	0.14 (0.05-0.31)
Poverty	30.3 (28.3-32.2)	30.0 (28.1-31.8)	30.8 (28.9-32.6)	29.3 (27.3-31.2)	26.1 (23.8-28.5)	34.6 (32.2-37.1)
Poverty plus at least 1 family adversity	4.3 (3.8-4.8)	5.7 (5.0-6.4)	5.7 (5.2-6.3)	5.3 (4.7-6.1)	6.5 (5.3-7.6)	8.6 (7.4-9.8)

Note – Data are % (95% CI – Clopper-Pearson), weighing variables: pttype2 (stratum variable), sptn00 (clustering at ward level), nh2 (finite population correction factor), survey weight ((aovwt2 (age 9 months), (bovwt2 (age 3), (covwt2 (age 5), (dovwt2 (age 7), (eovwt2 (age 11), (fovwt2 (age 14)).

Poor parental mental health: For the first survey (child aged 9 months*), the Rutter Malaise Inventory was used.

### Exposure Trajectories

**Figure 2:**
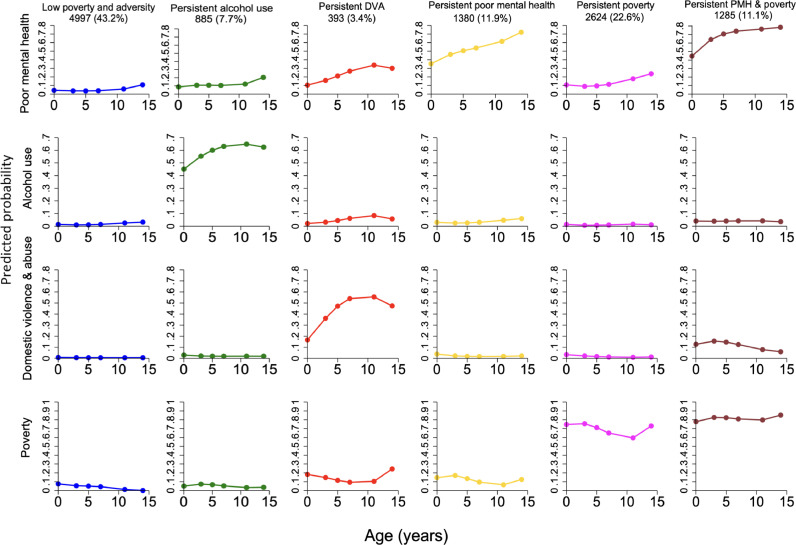
Estimated trajectory groups of family adversity and poverty in the UK Millennium Cohort Study.

Overall, the six-group trajectory model had the best fit (Fig. 2). The low poverty and adversity group comprised 4997 (43·2%) of children. The second largest group was “persistent poverty” comprising 2624 children (22·6%) with a high probability of poverty throughout childhood. A “persistent poor parental mental health” group comprised 1380 children (11·9%), characterised by high probability of exposure to poor parental mental health over time. A smaller percentage of children experienced persistent parental alcohol use (7·7%) and persistent parental domestic violence and abuse (3·4%). A “persistent poverty and poor parental mental health” group was evident comprising 11·1% of children who were more likely to experience co-occurrence of persistent poverty and poor parental mental health throughout childhood.

The characteristics of the cohort participants by the six estimated trajectory groups are shown in table 2 (see imputed dataset in appendix, pp 6). Co-occurrence of persistent poverty and poor parental mental health was more common among children of mothers with no educational qualifications. There were also differences by ethnicity, for example 19% of children in the persistent poverty and mental health group had Pakistani or Bangladeshi mothers compared to 1% in the low poverty and adversity group.

**Table 2:** Baseline characteristics and child health outcomes by the six estimated trajectory groups, observed data, weighted sample

	Predicted family adversity and poverty trajectories					
Characteristics	Low poverty and adversity (n=4997)	Persistent alcohol use (n=885)	Persistent domestic violence and abuse (n=393)	Persistent poor mental health (n=1380)	Persistent poverty (n=2624)	Persistent poverty and poor mental health (n=1285)
Female	2414 (48.3%)	436 (49.3%)	178 (45.3%)	663 (48.0%)	1318 (50.2%)	561 (43.7%)
Missing	115 (2.3%)	29 (3.2%)	6 (1.5%)	44 (3.2%)	137 (5.2%)	85 (6.6%)
Maternal education						
Degree plus	1382 (27.7%)	366 (41.4%)	76 (19.3%)	251 (18.2%)	55 (2.1%)	17 (1.3%)
Diploma	643 (12.9%)	101 (11.4%)	55 (14.0%)	128 (9.3%)	75 (2.8%)	24 (1.9%)
A-levels	625 (12.5%)	83 (9.4%)	55 (14.0%)	151 (10.9%)	138 (5.3%)	46 (3.6%)
GCSE A-C	1610 (32.2%)	220 (24.9%)	128 (32.6%)	505 (36.6%)	798 (30.4%)	358 (27.9%)
GCSE D-G	314 (6.3%)	39 (4.4%)	36 (9.2%)	146 (10.6%)	378 (14.4%)	196 (15.3%)
None	304 (6.1%)	47 (5.3%)	37 (9.4%)	153 (11.1%)	1034 (39.4%)	550 (42.8%)
Missing	119 (2.3%)	29 (3.2%)	6 (1.4%)	46 (3.3%)	146 (5.6%)	94 (7.3%)
Maternal ethnicity						
White	4504 (90.1%)	838 (94.7%)	334 (85.0%)	1167 (84.5%)	1677 (63.9%)	814 (63.3%)
Mixed	22 (0.4%)	6 (0.7%)	7 (1.8%)	11 (0.8%)	40 (1.5%)	19 (1.5%)
Indian	137 (2.7%)	2 (0.2%)	18 (4.6%)	40 (2.9%)	72 (2.7%)	29 (2.3%)
Pakistani and Bangladeshi	47 (1.0%)	0 (0%)	7 (1.8%)	36 (2.6%)	494 (18.8%)	244 (19.0%)
Black or Black British	98 (2.0%)	5 (0.6%)	15 (3.8%)	33 (2.4%)	146 (5.6%)	59 (4.6%)
Other ethnic groups	65 (1.3%)	4 (0.4%)	6 (1.5%)	45 (3.3%)	50 (1.9%)	31 (2.4%)
Missing	124 (2.5%)	30 (3.4%)	6 (1.5%)	48 (3.5%)	145 (5.5%)	89 (6.9%)
Socioemotional behavioural problems	218 (4.4%)	42 (4.8%)	51 (13.0%)	182 (13.2%)	289 (11.0%)	338 (26.3%)
Missing	123 (2.5%)	34 (3.8%)	10 (2.5%)	42 (3.0%)	123 (4.7%)	63 (4.9%)
Cognitive disability	221 (4.4%)	34 (3.8%)	19 (4.8%)	74 (5.4%)	250 (9.5%)	147 (11.5%)
Missing	347 (7.0%)	60 (6.8%)	28 (7.1%)	112 (8.1%)	242 (9.2%)	130 (10.1)
Obesity	241 (4.8%)	28 (3.2%)	24 (6.1%)	115 (8.3%)	254 (9.7%)	145 (11.3%)
Missing	256 (5.1%)	47 (5.3%)	13 (3.3%)	83 (6.0%)	225 (8.5%)	115 (8.9%)
Alcohol experimentation	2218 (44.4%)	487 (55.0%)	205 (52.2%)	610 (44.2%)	908 (34.6%)	462 (36.0%)
Missing	142 (2.8%)	30 (3.4%)	16 (4.0%)	68 (4.9%)	165 (6.3%)	101 (7.8%)
Drug experimentation	163 (3.3%)	45 (5.1%)	31 (7.9%)	51 (3.7%)	123 (4.7%)	85 (6.6%)
Missing	138 (2.8%)	30 (3.4%)	17 (4.3%)	69 (5.0%)	168 (6.4%)	106 (8.3%)

### Association between exposure trajectories and child health outcomes

**Figure 3:**
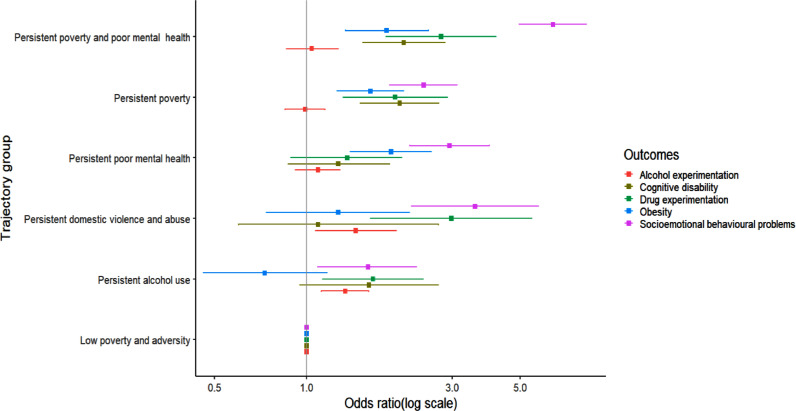
Associations of predicted family adversity and poverty trajectories and child outcomes at age 14 years in the UK Millennium Cohort Study. Models adjusted for child's sex, maternal education, and maternal ethnicity.

The associations of predicted trajectory groups and child outcomes at age 14 years are shown in Table 3 and figure 3. Both the adjusted and unadjusted ORs indicate that children in the five poverty and adversity groups, when compared with those in the low poverty and adversity group, have worse health outcomes at age 14 years, particularly child mental health. There was little association with child alcohol experimentation the odds of which were raised only for the parental alcohol use and DVA group. Any trajectory with high poverty was associated with over double the odds of poor child outcomes (with the exception of child alcohol experimentation). Associations were particularly strong between the clustering of persistent poverty and poor parental mental health and child outcomes. For example, compared with children with low poverty and adversity, those exposed to persistent poor parental mental health and poverty had higher odds of socioemotional behavioural problems (adjusted odds ratio 6·4; 95% CI 5·0 – 8·3), cognitive disability (aOR 2·1; CI 1·5 – 2·8), drug experimentation (aOR 2·8; CI 1·8 – 4·2) and obesity (aOR 1·8; CI 1·3 – 2·5). We did not find any significant association between this trajectory group and alcohol experimentation at age 14 years (OR: 1·0; 95% CI: 0·9 – 1·3). Adjustment for sex, maternal education, maternal ethnicity (Table 3, model 2) and lone parenthood (appendix, pp 9) resulted in slight attenuation of most associations when compared to the unadjusted estimates (see Table 3, model 1 vs. model 2). Sensitivity analysis using imputed data showed similar patterns of associations as the main analysis (appendix, pp 8). The sensitivity analysis using the multiple pseudo-class draw method^29^ to examine the effect of uncertainty that may arise from assigning trajectory group membership also showed similar results (appendix, pp 9) when compared to the maximum probability assignment rule.

**Table 3:** Associations of predicted family adversity and poverty trajectories and child outcomes at age 14 years in the UK Millennium Cohort Study

Odds ratio	Model*	Low poverty and adversity	Persistent alcohol use	Persistent domestic violence and abuse	Persistent poor mental health	Persistent poverty	Persistent poverty and poor mental health
Socioemotional behavioural problems (SDQ ≥17)	1	Ref.	1.45 (1.00-2.09)	3.84 (2.40-6.12)	3.26 (2.45-4.33)	3.05 (2.39-3.88)	8.45 (6.65-10.76)
	2	Ref.	1.59 (1.09-2.29)	3.56 (2.21-5.75)	2.94 (2.18-3.97)	2.42 (1.87-3.11)	6.41 (4.98-8.25)
Cognitive disability	1	Ref.	1.36 (0.81-2.28)	1.21 (0.69-2.11)	1.42 (0.97-2.06)	2.72 (2.06-3.60)	3.16 (2.35-4.23)
	2	Ref.	1.60 (0.95-2.70)	1.09 (0.60-2.70)	1.27 (0.87-1.87)	2.02 (1.50-2.71)	2.08 (1.53-2.84)
Alcohol experimentation	1	Ref.	1.40 (1.12-1.68)	1.35 (1.02-1.77)	1.04 (0.88-1.21)	0.74 (0.63-0.87)	0.84 (0.68-1.04)
	2	Ref.	1.34 (1.12-1.60)	1.45 (1.07-1.97)	1.09 (0.92-1.29)	0.99 (0.85-1.15)	1.04 (0.86-1.27)
Drug experimentation	1	Ref.	1.64 (1.12-2.39)	2.80 (1.50-5.22)	1.51 (1.03-2.21)	1.84 (1.32.2.53)	2.83 (2.00-3.99)
	2	Ref.	1.65 (1.13-2.41)	2.98 (1.62-5.47)	1.36 (0.89-2.05)	1.95 (1.32-2.89)	2.76 (1.82-4.18)
Obesity	1	Ref.	0.61 (0.39-0.98)	1.30 (0.75-2.21)	2.06 (1.52-2.78)	2.09 (1.65-2.65)	2.44 (1.84-3.24)
	2	Ref.	0.73 (0.46-1.17)	1.27 (0.75-2.17)	1.89 (1.39-2.56)	1.62 (1.26-2.08)	1.83 (1.34-2.51)

Note: SDQ – Strength and Difficulties Questionnaire.

⁎ Model 1- crude model; Model 2 – adjusted for child's sex, maternal education and maternal ethnicity.

## Discussion

Using the Millennium Cohort Study, a large nationally representative cohort sample of UK children, we showed that over half of children experienced trajectories of exposure to poverty and/or family adversity, which were associated with worse child physical, mental, cognitive and behavioural outcomes. Over 40% of children experienced high risk of exposure to either poverty and/or parental mental health problems throughout childhood. One in ten children experienced persistent risk of poverty and poor parental mental health up to age 14 which was associated with over six times the odds of child mental health problems, and double the odds of obesity and cognitive disability.

To our knowledge, this study is the first to assess the clustering of trajectories of child poverty and multiple indicators of family adversity and the impacts on subsequent child behaviour and health outcomes in adolescence in a representative UK sample. In a recent study in the UK, Lacey and colleagues^7^ used data from the Avon Longitudinal Study of Parents and Children (ALSPAC) to explore the clustering of childhood adversities. While that study also showed that poverty was strongly related to the clustering of multiple childhood adversities, the longitudinal experience of children had yet to be captured across their childhood - the central question in our current study using a nationally representative sample.

We identified six distinct trajectories of childhood family adversity and poverty; and found that almost all the high and persistent adversity trajectory groups were associated with substantially increased risk of adverse health behaviours and outcomes in early adolescence. Exposure to persistent poverty was a feature in two of the largest groups affecting around 30% of children. Exposure to parental mental health problems was also a feature of two of the groups, impacting around 20% or one in 5 children in this cohort. A smaller percentage of children experienced persistent parental alcohol use (7·7%) and persistent parental domestic violence and abuse (3·4%).

Our longitudinal results support a recent systematic review^30^ and a cross-sectional study^9^ suggesting that children may experience adversities in predictable combinations, leading to an increase in the risk of adverse health outcomes. Lanier and colleagues,^9^ for instance, used National survey of Children's Health data to investigate childhood adversities and found that parental mental ill health and poverty tend to co-occur or cluster. As previously observed,^8^ multiple clustered adversities can be particularly detrimental to children's health, and can lead to more enduring effects in later life compared to single adversities.^9^

In the UK there has been a policy focus on the potential clustering of three family level adversities – parental mental ill health, DVA and substance use, ^31^ as the prevalence of parental mental illness is rising over time. For example, a study by Abel and colleagues^5^ in the UK showed that maternal mental illness increased with age of child, from 21.9% of the youngest children (0–<2 years) to 27.3% in the oldest (14–<16 years), consistent with our findings on child-level prevalence on parental mental illness. Our analysis however demonstrates the extremely low level of clustering of these adversities. The poor mental health trajectory group (almost 12%) did not cluster with DVA and alcohol use. This may be due to the low prevalence of DVA (i.e. which does not include psychological and emotional violence) and alcohol use in the MCS which may also reflect how these risk factors were measured. Nevertheless, our findings corroborate other reports and reviews^1^^,^^31^; for example, Skinner and colleagues^31^ systematically reviewed the relationship between the so-called ‘toxic trio’ and found little evidence of the clustering of these risk factors. We, however, observed that both persistent parental DVA and alcohol use in isolation were associated with worse child outcomes. While we did not find any evidence of clustering of the so-called ‘toxic trio’ in this study, parental mental ill health has been shown to be linked with both parental alcohol use and DVA. ^7^ It remains possible, as we note that “persistent parental domestic violence and abuse” group also experience a moderately high probability of poor parental mental health.

The most important finding of our study is the clustering of the two most common exposures, poverty and poor parental mental health; and the synergistic impact of these exposures on child health. Over 40% of children were in trajectories with persistently high exposure to poverty and/or poor parental mental health, each exposure in isolation associated with a doubling of the odds of poor child mental and physical health. Children exposed to high levels of both poverty and poor parental mental health (11·1%) had over six times the odds of mental health problems compared with children in low adversity group. The link between mental health problems and poverty is well established,^23^^,^^32^ and the negative impact of their co-occurrence on children is far-reaching. The co-occurrence of both risk factors can be attributed to several causes, including stress, gene-environment interaction and psychological pathways.^32^ Transition into poverty for a family or household leads to increased risk of psychological distress and depression.^23^ This could partly explain the co-occurrence of poverty and poor parental mental health. There is also evidence that individuals with mental health problems are more likely to experience poverty subsequently – for example due to becoming unemployed as a result of their mental ill health – the social selection hypothesis.^32^ Our findings add to the current body of evidence by showing that poor parental mental health and poverty cluster and their persistence across the developmental stages is associated with poorer child health outcomes. From a syndemic systems perspective^15^ it is particularly important to further unpick the clustering of poverty and family mental health,^31^ and how they shape health inequalities across the lifecourse.

A key strength of the study is that we used the most contemporary national representative UK birth cohort, so we believe our findings are generalizable to the UK population, and particularly relevant for UK policy. Further research is needed to understand how trajectories of these adversities are experienced and cluster in other populations. There are very different levels of child poverty and parental mental health problems across different contexts^24^ and we would expect this to lead to different clustering patterns across contexts. However, in a Danish population-based cohort study, Rod and colleagues^8^ found similar groupings. For example, they found a trajectory group of “low adversity” (54%) and a trajectory group of “persistent material deprivation” (13%).” Another strength is that we used multiple imputation to account for missing data on outcomes and covariates. The application of a rigorous modelling technique to predict child developmental trajectories is also a strength of our study. This enabled us to estimate multiple risks over time whose existence has important implication for policy.

Despite these strengths, this study should be interpreted in the context of the following limitations. First, an underlying assumption of our modelling technique is that conditional on class membership, observations within classes are independent, essentially assuming that all individuals within a group follow the same trajectory. It is unlikely that this is truly the case and there may be variation in children's trajectories that we were not able to capture. This may have led to bias in the estimated class specific regression coefficients and reduced standard errors.^33^ However, we do not believe that this has a major impact on our results as our final model had high ability to classify individuals into groups and our interest was less in the exact shape of the trajectories over time within classes but rather in the groups themselves and the association with later outcomes. Second, although we used validated measures for children's socioemotional behavioural problems (i.e., SDQ)^20^ and parental mental ill health (i.e., Kessler 6 scale),^18^ both tools could have been subjected to reporting bias or measurement error. In the MCS, both measures were reported by the same person (caregiver), and a change in caregivers’ mental health status or mood can affect the rating of SDQ scores. However, prior studies found good inter-rater agreement between parent and teacher versions of the SDQ.^34^ The measures of alcohol and drug experimentation in the MCS are brief single questions which are unlikely to identify children with persistent problematic substance use. Also, clinical diagnosis for parental mental ill health was not measured in the MCS. However, validation of the Kessler 6 has indicated that it can predict mental ill health.^35^ Although the Kessler 6 scale replaced the Rutter Malaise Inventory as the measure of parental mental illness from wave two onwards in the MCS, it has been shown that both scales have good reliability and validity.^18^^,^^36^ Third, paternal mental ill health has not been explored in this study. In a recent review,^37^ Stein and colleagues found both maternal and paternal mental health to be associated with socioemotional and behavioural development of children. Fourth, self-reported domestic violence may be prone to underreporting, which is likely to bias our findings. We used physical abuse (use of force in a relationship) to capture the existence of violence and abuse in a household, as there is no information on other forms of abuse (e.g., sexual, emotional and financial) in the MCS. Fifth, we used parental alcohol consumption as a measure of substance misuse because data on drug use were not available in some waves. The MCS relied on self-reported data on both frequency and quantity of alcohol use. Information on quantity was however not consistent between waves, so we only used questions on frequency of alcohol use, as was done in previous studies.^38^ We have shown that the strong association between family adversity, poverty and child physical, mental, cognitive and behavioural outcomes largely persisted after adjustment for potential confounding by socio-demographic characteristics. However, data about potentially unmeasured confounding factors such as genetic risk factors were not included in our analysis. Although our study cannot ascertain whether the association between family adversity, poverty and child outcomes is causal, the strength of the effect sizes, and coherence with previous studies suggest that it may be.^39^ A systematic reviews of quasi-experimental studies supports a causal effect of income on child emotional and behavioural outcomes.^40^ However, a recent study by Sariaslan and colleagues^41^ used a sibling design in population linked data in Finland, showing that the observed association between family income in childhood and risk of psychiatric disorders in later life was explained by family level factors, with attenuation of effects in the sibling analysis. The authors suggest that further large-scale studies with long-term follow up are required, while noting that RCTs of anti-poverty interventions have been effective in some settings.

Nevertheless, this study is the first to explore the clustering of trajectories of family adversity and child poverty across the early life course, as opposed to a single adversity trajectory^10^ and accumulation of risk approach.^7^ Although our life-course approach and the accumulation of risk are highly intertwined,^8^ the latter does not consider patterns of children's risk exposure over time (i.e., sensitive and critical periods of exposure).

Our longitudinal analysis provides strong evidence that adverse conditions have important effects on children's lives, but it is even more detrimental when multiple risk factors co-occur. The few previous studies on trajectories of childhood adversity looked at single key risk exposures such as maternal mental health.^42^ However, the fact that specific adversities may act syndemically,^8^ often with structural risk factors such as poverty,^9^ as was found in this study, has important implications for public policies and interventions.

Our findings show that interventions to address specific childhood adversities may not be meaningful if childhood socioeconomic conditions such as poverty are not considered. These findings are crucial to the ongoing discussion about incorporating an understanding of syndemics in clinical and public health,^15^^,^^42^ as the complex interplay of large-scale social forces, biomedical factors and childhood adversities may shape health inequalities. The syndemic approach acknowledges that health risk and problems tend to cluster among individuals who are already vulnerable because of economic conditions and other structural factors. Therefore, interventions that incorporate the concept of syndemics are likely to be more effective and implementable than one risk or one problem interventions.^43^

It is now vital that policies to “level-up” in the post COVID recovery focus on protecting the next generation from the adversities that cluster with poverty. Parental alcohol use and DVA in the UK are also growing issues,^4^ and undoubtedly important factors in children's lives, but affect fewer children than poverty and mental health problems.^5^^,^^10^^,^^23^ In the UK both parental mental ill health^5^ and child poverty^44^ are rising. One in three children in the UK is in poverty and, currently, one in six children and young people have mental health problems, with clear implications for long term life chances.^45^ Although the relation between household poverty and parental mental health is likely to be complex, poverty is an easily modifiable risk factor.^10^^,^^19^ In the UK, immediate policy considerations include retaining the universal credit uplift and reversing changes to the welfare system that have led to rising child poverty; re-investing in support services and children's preventive services such as children's centres; and improving access to mental health services for families.

### Ethics approval and consent to participate

Ethical approval for each wave of the MCS was granted by NHS Multicenter Research Ethics Committees. No further ethical approval was required for this secondary analysis of MCS data.

### ORACLE consortium members

Rebecca Lynch, Simon Barrett, Samantha Burns, Sarwar Tubah, Julia Forman, Raeena Hirve, Mary Bangisky, Harriet Boulding, Simon Hackett and Julia Fox-Rushby.

## Contributors

NKA carried out the statistical analyses and led the drafting of the manuscript (supported by DTR). NKA, DTR, DKS, KMF, VSS and GM contributed to the study design and analysis plan. NKA, DTR, DKS, KMF, VSS, GM, RM, LH, EK and IW contributed to the conception of the study, interpretation of results and critically reviewed the manuscript for its intellectual content. NKA, DTR, DKS and KMF accessed and verified the data reported in the study. All authors approved the final manuscript as submitted and agree to be accountable for all aspects of the work.

## Declaration of interests

This work was funded by the National Institute for Health Research (NIHR) Policy Research Programme (ORACLE: OveRcoming Adverse ChiLdhood Experiences, Grant reference number NIHR200717); and the National Institute for Health Research (NIHR) Applied Research Collaboration South London (NIHR ARC South London) at King's College Hospital NHS Foundation Trust. DTR is supported by the NIHR School for Public Health Research, the NIHR Public Health Policy Research and by the Medical Research Council (MRC) on a Clinician Scientist Fellowship (MR/P008577/1). VSS is supported by the Swedish Research Council for Health, Working Life and Welfare (FORTE) [2016-07148; 2020-00274]. DKS is supported by the FORTE [2020-00274] and the NIHR Public Health Policy Research Unit. Professors Kaner and Howard are supported by NIHR Senior Investigator awards and Prof Kaner is Director of the NIHR Applied Research Collaboration for the North East and North Cumbria. The views expressed in this publication are those of the authors and not necessarily those of the NIHR, MRC or FORTE. All authors declare no competing interests.

## Appendix Supplementary materials

Image, application 1

Image, application 2
