# Supplementary material for: Impact of poverty and family adversity on adolescent health: a multi-trajectory analysis using the UK Millennium Cohort Study
Source: Lancet Reg Health Eur. 2021 Nov 30;13:100279. doi: 10.1016/j.lanepe.2021.100279 (PMC8841277; doi:10.1016/j.lanepe.2021.100279)
Supplement: Supplementary file 1 [file mmc1.docx]

**Supplementary Appendix**
