# Supplementary material for: Impact of poverty and family adversity on adolescent health: a multi-trajectory analysis using the UK Millennium Cohort Study
Source: Lancet Reg Health Eur. 2021 Nov 30;13:100279. doi: 10.1016/j.lanepe.2021.100279 (PMC8841277; doi:10.1016/j.lanepe.2021.100279)
Supplement: Supplementary file 2 [file mmc2.docx]

**Supplementary Box 1.** Search terms

We systematically searched MEDLINE, PsycInfo, and the Web of Science for articles published up to March 15, 2021, without language restrictions for studies that assessed the associations between family adversity (measured using poor parental mental health, domestic violence and abuse and parental alcohol use) with the search terms ( “mental health”, “mental ill health”, “wellbeing”, “emotional instability”, “anxiety”, “anxious”, “depression”, “depressive”) OR (“domestic”, “partner”, “family”, “violence”, “abuse”) OR (“alcohol use”, “substance use”, “abuse”, “addiction”, “alcoholism”), poverty with the search terms (“poverty”, “poor“, “deprived”, “deprivation”, “indigence”, “indigent”, “impoverished”, “impoverishment”, “destitute”, “destitution”, “SES” , “socio-economic status”, “inequalities”); as well as relevant umbrella terms (“toxic trio”, “trigger trio”, “child adversity”, “family adversity”, “stressors”) and adolescent health outcomes with the search terms (“health outcomes”, “outcomes”, “adolescent”, “adolescence”, “teen”, “juvenile”, “youth”).


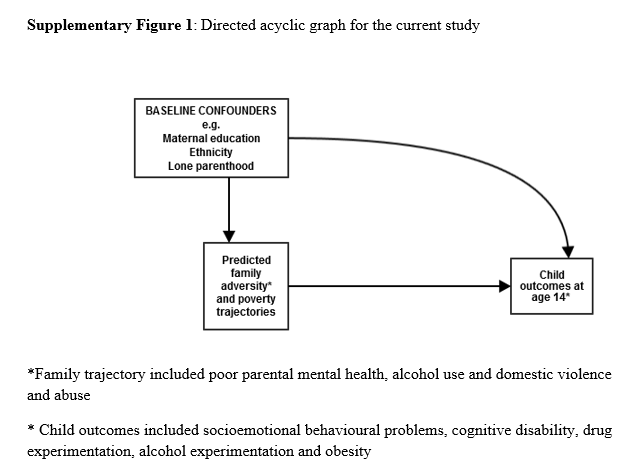


**Technical appendix.** Group-based multi-trajectory model

The two key outputs of the GBTM applied in this paper are the shape of the trajectory, defined by a cubic function of age, and the individual’s probability of trajectory group membership.^1^ We ran the models in Stata via the package TRAJ^1^ and used logistic regression to model the conditional probabilities of individuals’ states for binary outcomes over time.

The model yields a probability for each individual of being in each trajectory group, as follows.

For each individual *i*, let

$\boldsymbol{Y}_{i}^{1}$ denote the vector of binary state of Mental ill health at ages 9 months, 3, 5, 7, 11 and 14 years

$\boldsymbol{Y}_{i}^{2}$ denote the vector of binary state of DVA at ages 9 months, 3, 5, 7, 11 and 14 years,

$\boldsymbol{Y}_{i}^{3}$ denote the vector of binary state of Alcohol use at ages 9 months, 3, 5, 7, 11 and 14 years,

$\boldsymbol{Y}_{i}^{4}$ denote the vector of binary state of Poverty at ages 9 months, 3, 5, 7, 11 and 14 years, and

$j_{i} \in(1, \ldots, 6)$ denotes the latent trajectory groups identified for our final model.

The group-based multi-trajectory model assumes that subjects belong to a trajectory group, *j*, with the following likelihood function for each individual *i*

$$Pr( \boldsymbol{Y}_{i}^{1}=\boldsymbol{y}_{i}^{1}, \ldots, \boldsymbol{Y}_{i}^{4}=\boldsymbol{y}_{i}^{4})=$$

$$\sum_{j=1}^{6}Pr(J_{i}=j)Pr\left( \boldsymbol{Y}_{i}^{1}=\boldsymbol{y}_{i}^{1}, \ldots, \boldsymbol{Y}_{i}^{4}=\boldsymbol{y}_{i}^{4} | J_{i}=j \right)$$

Where the latter factor is the probability of the longitudinal data for subject i given that this subject belongs to trajectory group j. This probability is given by

$$\Pr\left( \boldsymbol{Y}_{i}^{1}=\boldsymbol{y}_{i}^{1}, \ldots, \boldsymbol{Y}_{i}^{4}=\boldsymbol{y}_{i}^{4} | J_{i}=j \right)=$$

$\Pr\left( \boldsymbol{Y}_{i}^{1}=\boldsymbol{y}_{i}^{1}{|J}_{i}=j)Pr( \boldsymbol{Y}_{i}^{2}=\boldsymbol{y}_{i}^{2} | J_{i}=j \right)\ldots Pr\left( \boldsymbol{Y}_{i}^{4}=\boldsymbol{y}_{i}^{4} | J_{i}=j \right)$Following from the equations above, which define conditional independence of the indicators $\boldsymbol{Y}^{1}$ to $\boldsymbol{Y}^{\boldsymbol{4}}$ once the trajectory groups are identified, the model is developed as follows. Let our unobservable discrete variable *J_i_* indicate the latent trajectory of the *i-th* individual. *J_i_* is assumed to take on *J* values, 6 in our case, each corresponding to a distinct expected trajectory *j*. Our model follows a finite mixture distribution of order J with *K* outcome components where the likelihood for each individual conditional on number of groups *J* may be written as

$$P\left( Y_{i}^{1}, \ldots, Y_{i}^{k}|{Age}_{i} \right)= \sum_{j=1}^{J} Pr(J_{i}=j)\prod_{k=1}^{K} P_{k}\left( Y_{i}^{k} \right|{Age}_{i}, j; \beta_{j}^{k})$$

With

$${P_{k}\left( Y_{i}^{k} \right|Age}_{i}, j; \beta_{j}^{k})=\prod_{t=1}^{T^{k}} p_{k}\left( y_{it}^{k} \right| {age}_{it}, j; \beta_{j}^{k})$$

where each of the *k* indicators can be measured at different time points *T = t*. Here the conditional distribution of $Y_{i}^{k}$, given membership in *j* is indexed by the unknown parameter vector $\beta_{j}$ which determines the shape of the group-specific trajectory. We estimated $p_{k}(*)$ using a logistic regression model with a cubic function of age for all *k*.

The equations highlight the assumed independence between measurements at different time points within individuals conditional on group membership.

**Supplementary Table 1**. Model selection Results

| Number of groups | BIC | AIC |
| --- | --- | --- |
| 1 | -85823.59 | -85740.47 |
| 2 | -74515.56 | -74344.13 |
| 3 | -72096.04 | -71836.30 |
| 4 | -69195.50 | -68847.44 |
| 5 | -68265.32 | -67828.95 |
| 6 | -67868.45 | -67343.76 |
| 7 | -67767.82 | -66954.80 |

Note. BIC – Bayesian information criterion; AIC – Akaike information criterion

**Supplementary Table 2**. Model Adequacy Results

| Trajectory group | N | A*ve*PP | OCC |
| --- | --- | --- | --- |
| Low poverty and adversity | 4997 (43.2%) | 0.92 | 16 |
| Persistent alcohol use | 885 (7.7%) | 0.92 | 139 |
| Persistent domestic violence and abuse | 393 (3.4%) | 0.84 | 145 |
| Persistent poor mental health | 1380 (11.9%) | 0.81 | 32 |
| Persistent poverty | 2624 (22.6%) | 0.86 | 21 |
| Persistent poverty and mental ill health | 1285 (11.1%) | 0.85 | 48 |

Note. A*ve*PP - average posterior probability; OCC – odds of correct classification.

Membership probability greater than 0.70 and OCC greater than 5 represent a good model fit

**Supplementary Table 3**. Weighted number of families in adversity and poverty in each wave of follow up

|  |  |  |  |  |  |  |
| --- | --- | --- | --- | --- | --- | --- |
| Follow-up time | Age 9 months | Age 3 | Age 5 | Age 7 | Age 11 | Age 14 |
|  | Weighted count (%) | Weighted count (%) | Weighted count (%) | Weighted count (%) | Weighted count (%) | Weighted count (%) |
| Domestic violence and abuse | 476 (3.6) | 463 (4.4) | 421 (4.0) | 353 (3.8) | 347 (3.9) | 234 (3.2) |
| Parental alcohol use | 758 (5.5) | 830 (6.7) | 914 (7.8) | 873 (7.4) | 990 (8.3) | 856 (7.7) |
| Poor parental mental health | 2615 (13.6) | 2641 (19.0) | 2736 (18.8) | 2525 (20.0) | 3200 (28.3) | 3182 (32.1) |
| Poverty | 6769 (30.3) | 5029 (30.0) | 4353 (29.0) | 4110 (29.3) | 3272 (26.1) | 3418 (34.6) |

Note. weighing variables: pttype2 (stratum variable), sptn00 (clustering at ward level), nh2 (finite population correction factor), survey weight ((aovwt2 (age 9 months), (bovwt2 (age 3), (covwt2 (age 5), (dovwt2 (age 7), (eovwt2 (age 11), (fovwt2 (age 14)). Poor parental mental health: For the first survey (child aged 9 months*), the Rutter Malaise Inventory was used.

**Supplementary Table 4.** Baseline characteristics of the cohort participants by the six estimated trajectory groups, imputed data

|  |  |  |  |  |  |  |
| --- | --- | --- | --- | --- | --- | --- |
|  | **Predicted family adversity and poverty trajectories** | | | | | |
| Characteristics | Low poverty and adversity (n=4997) | Persistent alcohol use (n=885) | Persistent domestic violence and abuse (n=393) | Persistent poor mental health (n=1380) | Persistent poverty (n=2624) | Poverty and poor mental health (n=1285) |
|  | % | % | % | % | % | % |
| Child's sex |  |  |  |  |  |  |
| Male | 50.6 | 48.8 | 54.5 | 50.4 | 47.2 | 53.5 |
| Female | 49.4 | 51.2 | 45.5 | 49.6 | 52.8 | 46.5 |
| Maternal education |  |  |  |  |  |  |
| Degree plus | 28.3 | 42.5 | 19.6 | 18.5 | 2.3 | 1.6 |
| Diploma | 13.2 | 11.9 | 14.5 | 9.5 | 3.0 | 1.9 |
| A-levels | 12.7 | 9.9 | 13.9 | 11.3 | 5.7 | 3.9 |
| GCSE A-C | 33.1 | 25.7 | 32.6 | 37.8 | 32.7 | 29.9 |
| GCSE D-G | 6.5 | 4.6 | 9.7 | 10.9 | 15 | 16.5 |
| None | 6.2 | 5.4 | 9.7 | 12 | 41.3 | 46.2 |
| Maternal ethnicity |  |  |  |  |  |  |
| White | 92.4 | 98 | 86.2 | 87.3 | 67.8 | 67.2 |
| Mixed | 0.5 | 0.7 | 1.8 | 1.0 | 1.7 | 1.9 |
| Indian | 2.8 | 0.2 | 4.6 | 2.9 | 2.8 | 2.3 |
| Pakistani and Bangladeshi | 1.0 | 0 | 1.8 | 2.8 | 20 | 20.9 |
| Black or Black British | 2.0 | 0.7 | 4.1 | 2.7 | 5.8 | 4.9 |
| Other ethnic groups | 1.3 | 0.4 | 1.5 | 3.3 | 1.9 | 2.8 |
| Socioemotional behavioural problems |  |  |  |  |  |  |
| SDQ score <17 | 95.5 | 94.9 | 87 | 86.3 | 88.2 | 72.8 |
| SDQ score ≥17 | 4.5 | 5.1 | 13 | 13.7 | 11.8 | 27.2 |
| Cognitive disability |  |  |  |  |  |  |
| No | 95.1 | 95.9 | 94.9 | 93.3 | 89.6 | 86.8 |
| Yes | 4.9 | 4.1 | 5.1 | 6.7 | 10.4 | 13.2 |
| Obesity |  |  |  |  |  |  |
| Not obese | 95.0 | 96.7 | 93.6 | 91.1 | 89.3 | 87.3 |
| Obese | 5.0 | 3.3 | 6.4 | 8.9 | 10.7 | 12.7 |
| Alcohol experimentation |  |  |  |  |  |  |
| No | 54.1 | 43.2 | 46.6 | 53.5 | 62.6 | 60.5 |
| Yes | 45.9 | 56.8 | 53.4 | 46.5 | 37.4 | 39.5 |
| Drug experimentation |  |  |  |  |  |  |
| No | 96.6 | 94.6 | 91.9 | 96.1 | 94.9 | 92.7 |
| Yes | 3.4 | 5.4 | 8.1 | 3.9 | 5.1 | 7.3 |

_Note. Only percentages are presented for multiply imputed data as the_ *_ns_* _vary across the 25 imputed dataset*_

_Multiple imputation by chained equation with pooled results using Rubins rules_^2^

**Supplementary Table 5.** Associations of predicted family adversity and poverty trajectories and child outcomes at age 14 years in the UK Millennium Cohort Study, imputed data^¥^

|  |  |  |  |  |  |  |  |
| --- | --- | --- | --- | --- | --- | --- | --- |
| Odds ratio | **Model*** | Low poverty and adversity | Persistent alcohol use | Persistent domestic violence and abuse | Persistent poor mental health | Persistent poverty | Persistent poverty and poor mental health |
| Socioemotional behavioural problems (SDQ ≥17) | 1 | Ref. | 1.40 (0.97-2.03) | 3.79 (2.38-6.02) | 3.26 (2.46-4.33) | 3.06 (2.39-3.93) | 8.23 (6.47-10.46) |
|  | 2 | Ref. | 1.53 (1.05-2.21) | 3.53 (2.19-5.68) | 2.96 (2.20-4.00) | 2.46 (1.90-3.20) | 6.29 (4.86-8.13) |
| Cognitive disability | 1 | Ref. | 1.32 (0.80-2.18) | 1.23 (0.68-2.22) | 1.45 (1.00-2.10) | 2.68 (2.03-3.55) | 3.18 (2.37-4.27) |
|  | 2 | Ref. | 1.55 (0.93-2.60) | 1.10 (0.59-2.07) | 1.30 (0.88-1.92) | 1.97 (1.46-2.66) | 2.10 (1.52-2.90) |
| Alcohol experimentation | 1 | Ref. | 1.38 (1.15-1.66) | 1.33 (1.01-1.75) | 1.03 (0.88-1.21) | 0.75 (0.63-0.89) | 0.83 (0.67-1.02) |
|  | 2 | Ref. | 1.32 (1.10-1.58) | 1.43 (1.06-1.94) | 1.07 (0.91-1.27) | 0.99 (0.85-1.16) | 1.04 (0.85-1.26) |
| Drug experimentation | 1 | Ref. | 1.59 (1.08-2.34) | 2.72 (1.48-5.01) | 1.50 (1.03-2.18) | 1.84 (1.33.2.55) | 2.79 (1.98-3.94) |
|  | 2 | Ref. | 1.60 (1.09-2.35) | 2.88 (1.58-5.24) | 1.35 (0.90-2.03) | 1.94 (1.32-2.85) | 2.67 (1.76-4.05) |
| Obesity | 1 | Ref. | 0.64 (0.39-1.05) | 1.31 (0.76-2.27) | 2.03 (1.50-2.75) | 2.10 (1.65-2.69) | 2.45 (1.85-3.25) |
|  | 2 | Ref. | 0.75 (0.46-1.24) | 1.29 (0.75-2.22) | 1.86 (1.37-2.53) | 1.64 (1.26-2.14) | 1.87 (1.36-2.56) |

Note: * Model 1- crude model; Model 2 – adjusted for child’s sex, maternal education and maternal ethnicity

SDQ – Strength and Difficulties Questionnaire

^¥^ Multiple imputation by chained equation (25 imputed datasets) with results pooled using Rubins’s rules^2^

**Supplementary Table 6.** Associations of predicted family adversity and poverty trajectories and child outcomes at age 14 years in the UK Millennium Cohort Study

|  |  |  |  |  |  |  |
| --- | --- | --- | --- | --- | --- | --- |
| Odd ratio* | Low poverty and adversity | Persistent alcohol use | Persistent domestic violence and abuse | Persistent poor mental health | Persistent poverty | Persistent poverty and poor mental health |
| Socioemotional behavioural problems (SDQ ≥17) | Ref. | 1.47 (1.00-2.18) | 3.67 (2.22-6.07) | 2.84 (2.10-3.85) | 2.24 (1.67-3.01) | 5.98 (4.47-7.99) |
| Cognitive disability | Ref. | 1.62 (0.94-2.80) | 1.06 (0.57-1.98) | 1.36 (0.93-2.01) | 2.11 (1.47-3.03) | 1.87 (1.27-2.72) |
| Alcohol experimentation | Ref. | 1.35 (1.13-1.61) | 1.42 (1.04-1.96) | 1.08 (0.92-1.28) | 0.86 (0.71-1.03) | 1.00 (0.77-1.29) |
| Drug experimentation | Ref. | 1.72 (1.16-2.53) | 3.13 (1.67-5.88) | 1.50 (0.98-2.30) | 1.28 (0.80-2.07) | 3.15 (1.90-5.21) |
| Obesity | Ref. | 0.76 (0.47-1.23) | 1.35 (0.78-2.33) | 1.85 (1.37-2.52) | 1.59 (1.18-2.15) | 1.51 (1.01-2.25) |

Note: * Model adjusted for child’s sex, maternal education, maternal ethnicity and lone parenthood.

**Supplementary Table 7.** Associations of predicted family adversity and poverty trajectories and child outcomes at age 14 years in the UK Millennium Cohort Study using the multiple pseudo-class draw method^¥^

|  |  |  |  |  |  |  |
| --- | --- | --- | --- | --- | --- | --- |
| Odds ratio* | Low poverty and adversity | Persistent alcohol use | Persistent domestic violence and abuse | Persistent poor mental health | Persistent poverty | Persistent poverty and poor mental health |
| Socioemotional behavioural problems (SDQ ≥17) | Ref. | 1.55 (0.95-2.53) | 3.17 (1.94-5.15) | 2.98 (1.69-5.25) | 2.47 (1.53-3.99) | 6.29 (4.78-8.27) |
| Cognitive disability | Ref. | 1.55 (0.84-2.87) | 1.18 (0.64-2.15) | 1.25 (0.83-1.87) | 2.01 (1.45-2.78) | 2.10 (1.46-3.00) |
| Alcohol experimentation | Ref. | 1.41 (1.08-1.84) | 1.39 (0.98-1.95) | 1.15 (0.95-1.40) | 1.01 (0.85-1.19) | 1.03 (0.84-1.27) |
| Drug experimentation | Ref. | 1.70 (1.02-2.83) | 2.80 (1.32-5.93) | 1.55 (0.98-2.44) | 2.02 (1.33-3.07) | 2.75 (1.77-4.27) |
| Obesity | Ref. | 0.78 (0.48-1.29) | 1.45 (0.79-2.66) | 1.80 (1.23-2.62) | 1.61 (1.13-2.28) | 1.76 (1.19-2.61) |

Note: * Model adjusted for child’s sex, maternal education and maternal ethnicity

SDQ – Strength and Difficulties Questionnaire

^¥^Sensitivity analysis using multiple pseudo class draw method^3^ (20 draws) to account for potential misclassification error

**Supplementary Figure 2.** Associations of predicted family adversity and poverty trajectories and child outcomes at age 14 years in the UK Millennium Cohort Study. Models adjusted for child’s sex, maternal education, and maternal ethnicity


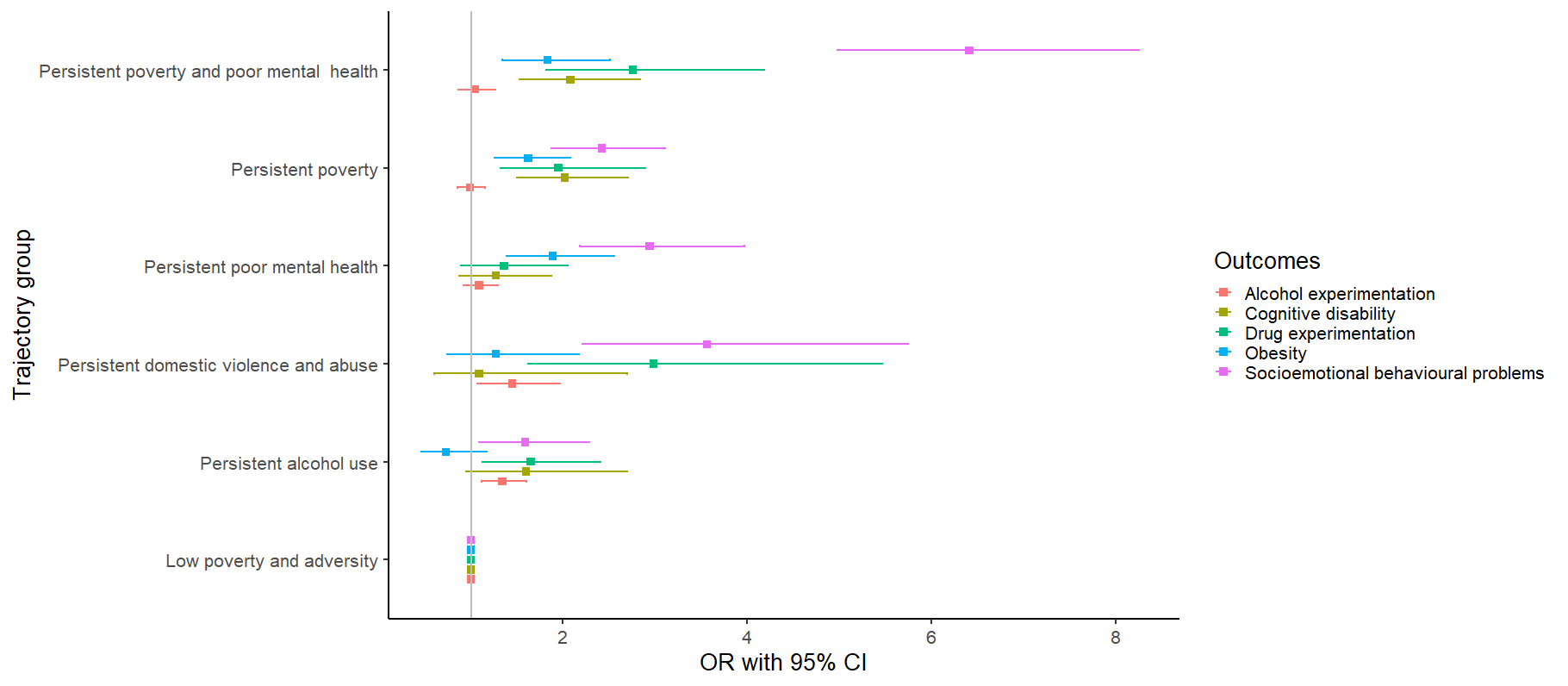


**Supplementary Table 7.** STROBE Statement—Checklist of items that should be included in reports of *cohort studies*

|  | **Item No** | **Recommendation** | **Location** |
| --- | --- | --- | --- |
| **Title and abstract** | 1 | (*a*) Indicate the study’s design with a commonly used term in the title or the abstract | Title |
|  |  | (*b*) Provide in the abstract an informative and balanced summary of what was done and what was found | Abstract – pp 1, line 8-22 |
| **Introduction** | | |  |
| Background/rationale | 2 | Explain the scientific background and rationale for the investigation being reported | Introduction – pp 3, paragraphs 1-3 |
| Objectives | 3 | State specific objectives, including any prespecified hypotheses | Introduction – pp 3 line 50-64 |
| **Methods** | | |  |
| Study design | 4 | Present key elements of study design early in the paper | Methods – pp 4,  line 67-80 |
| Setting | 5 | Describe the setting, locations, and relevant dates, including periods of recruitment, exposure, follow-up, and data collection | Methods – pp 4,  line 67-80 |
| Participants | 6 | (*a*) Give the eligibility criteria, and the sources and methods of selection of participants. Describe methods of follow-up  (*b*) For matched studies, give matching criteria and number of exposed and unexposed | Methods – pp 4,  line 70-80, Figure 1  N/A |
| Variables | 7 | Clearly define all outcomes, exposures, predictors, potential confounders, and effect modifiers. Give diagnostic criteria, if applicable | Methods – pp 4-5,  line 90-123,  box 1 |
| Data sources/ measurement | 8* | For each variable of interest, give sources of data and details of methods of assessment (measurement). Describe comparability of assessment methods if there is more than one group | Methods – pp 4,  line 67-79 |
| Bias | 9 | Describe any efforts to address potential sources of bias | Methods -pp 6, line 145, line 149-155 |
| Study size | 10 | Explain how the study size was arrived at | Methods -pp 4, line 74-79 |
| Quantitative variables | 11 | Explain how quantitative variables were handled in the analyses. If applicable, describe which groupings were chosen and why | Methods -pp 4, line 90-94  box 1 |
| Statistical methods | 12 | (*a*) Describe all statistical methods, including those used to control for confounding | Methods – pp 5-6, line 125-155 |
|  |  | (*b*) Describe any methods used to examine subgroups and interactions | Methods - pp 6, line 153-155 |
|  |  | (*c*) Explain how missing data were addressed | Methods - pp 6, line 150-152 |
|  |  | (*d*) If applicable, describe analytical methods taking account of sampling strategy | Methods - pp 6, line 143-45 |
|  |  | (*e*) Describe any sensitivity analyses | Methods - pp 6, line 149-155 |
| **Results** | | |  |
| Participants | 13* | (a) Report numbers of individuals at each stage of study—eg numbers potentially eligible, examined for eligibility, confirmed eligible, included in the study, completing follow-up, and analysed | Results – pp 7, line 163-166  Figure 1 |
|  |  | (b) Give reasons for non-participation at each stage | Figure 1 |
|  |  | (c) Consider use of a flow diagram | Figure 1 |
| Descriptive data | 14* | (a) Give characteristics of study participants (eg demographic, clinical, social) and information on exposures and potential confounders | Results – pp 7 line 185-190, Table 3 |
|  |  | (b) Indicate number of participants with missing data for each variable of interest  (c) Summarise follow-up time (eg, average and total amount) | Table 3 |
| Outcome data | 15* | Report numbers of outcome events or summary measures | Table 4 |
| Main results | 16 | (*a*) Give unadjusted estimates and, if applicable, confounder-adjusted estimates and their precision (eg, 95% confidence interval). Make clear which confounders were adjusted for and why they were included | Table 4 |
|  |  | (*b*) Report category boundaries when continuous variables were categorized | N/A |
|  |  | (*c*) If relevant, consider translating estimates of relative risk into absolute risk for a meaningful time period | N/A |
| Other analyses | 17 | Report other analyses done—eg analyses of subgroups and interactions, and sensitivity analyses | Results – pp 8, line 209-213 |
| **Discussion** | | |  |
| Key results | 18 | Summarise key results with reference to study objectives | Discussion – pp 8, line 216-239 |
| Limitations | 19 | Discuss limitations of the study, taking into account sources of potential bias or imprecision. Discuss both direction and magnitude of any potential bias | Discussion – pp 11-12, line 296-340 |
| Interpretation | 20 | Give a cautious overall interpretation of results considering objectives, limitations, multiplicity of analyses, results from similar studies, and other relevant evidence | Discussion – pp 12-13 line 346-372 |
| Generalisability | 21 | Discuss the generalisability (external validity) of the study results | Discussion – pp 10 line 284-292 |
| **Other information** | | |  |
| Funding | 22 | Give the source of funding and the role of the funders for the present study and, if applicable, for the original study on which the present article is based | pp 14, line 389-400 |

*Give information separately for exposed and unexposed groups.

**Note:** An Explanation and Elaboration article discusses each checklist item and gives methodological background and published examples of transparent reporting. The STROBE checklist is best used in conjunction with this article (freely available on the Web sites of PLoS Medicine at http://www.plosmedicine.org/, Annals of Internal Medicine at http://www.annals.org/, and Epidemiology at http://www.epidem.com/). Information on the STROBE Initiative is available at www.strobe-statement.org.

**References**

1. Nagin DS, Jones BL, Passos VL, Tremblay RE. Group-based multi-trajectory modeling. *Statistical methods in medical research* 2018; **27**(7): 2015-23.

2. Rubin DB. Multiple imputation for nonresponse in surveys: John Wiley & Sons; 2004.

3. Bray BC, Lanza ST, Tan X. Eliminating bias in classify-analyze approaches for latent class analysis. *Structural equation modeling: a multidisciplinary journal* 2015; **22**(1): 1-11.
